# University Students’ Hangover May Affect Cognitive Research

**DOI:** 10.3389/fpsyg.2020.573291

**Published:** 2020-09-29

**Authors:** Mauro Murgia, Serena Mingolo, Valter Prpic, Fabrizio Sors, Ilaria Santoro, Eleonora Bilotta, Tiziano Agostini

**Affiliations:** ^1^Department of Life Sciences, University of Trieste, Trieste, Italy; ^2^Institute for Psychological Science, De Montfort University, Leicester, United Kingdom; ^3^Department of Physics, University of Calabria, Rende, Italy

**Keywords:** alcohol, hangover, cognition, internal validity, research methods

## Abstract

University students are the most employed category of participants in cognitive research. However, researchers cannot fully control what their participants do the night before the experiments (e.g., consumption of alcohol) and, unless the experiment specifically concerns the effects of alcohol consumption, they often do not ask about it. Despite previous studies demonstrating that alcohol consumption leads to decrements in next-day cognitive abilities, the potential confounding effect of hangover on the validity of cognitive research has never been addressed. To address this issue, in the present study, a test-retest design was used, with two groups of university students: at T0, one group was constituted by hungover participants, while the other group was constituted by non-hungover participants; at T1, both groups were re-tested in a non-hangover state. In particular, the tests used were two versions of a parity judgment task and an arithmetic verification task. The results highlight that: (a) the response times of university students experiencing a hangover are significantly slower than those of non-hangover students and (b) the response times of hungover students are slower than those of the same students when re-tested in a non-hangover state. Additionally, it was also observed that the prevalence of hungover students in the university campus varies depending on the day of the week, with a greater chance of enrolling hungover participants on specific days. In light of the latter result, the recruitment of university students as participants in cognitive experiments might lead researchers to erroneously attribute their results to the variables they are manipulating, ignoring the effects of the potential hangover state.

## Introduction

A large part of what we know about psychology derives from experiments run on university students and laboratory animals. When describing the studies on laboratory animals, researchers are usually very accurate in reporting the conditions under which their animals are kept. In particular, the environmental conditions (e.g., temperature, cages dimensions, and lighting) and the feeding (e.g., food and water administration) are strictly controlled and meticulously described (for an example, see Chiandetti et al., 2014). Conversely, when testing human participants, researchers make the implicit assumption that all their participants before starting an experiment are roughly in the same conditions, and that these conditions are optimal to serve as subjects (e.g., Corona et al., 2016; Guicciardi et al., 2019). However, the majority of researchers do not have full control over the behaviors of their participants in the 24 h before the experiments, and often they do not worry about it. This may represent an underestimated methodological issue, which could threaten the internal validity of experiments, since participants might be in psychophysiological conditions that could affect their performance.

In particular, a possible threat to the internal validity is the consumption of alcohol by university students, the night before their participation in cognitive experiments. Indeed, it is well-known that students tend to party and to consume large amounts of alcohol. It has been demonstrated that alcohol consumption among university students is higher compared to non-college age-mates (O’Malley and Johnston, 2002), and that students drink not only on weekends, but also on weekdays. In particular, it happens mostly on Wednesdays in the United Kingdom as anecdotally reported by Stephens et al. (2014), and on Thursdays as well as on days of celebrations, such as birthdays and campus/university events, in the United States (Del Boca et al., 2004; Glindemann et al., 2007), however, the preferred day of the week for student’s parties may vary depending on the city. After weekday parties, a certain percentage of students might decide to stay at home and recover, while others might still go to the university and carry out their daily duties. The latter group of students is potentially eligible for taking part to psychological experiments, especially if they had previously scheduled it in exchange of money or academic credits. However, the data deriving from their test might be biased by the combined effect of alcohol consumption and reduced sleep quality (Penning et al., 2010), which might impair their cognitive performances.

The cognitive effects associated with alcohol consumption have been largely investigated (Ryback, 1971; Koelega, 1995; Schweizer and Vogel-Sprott, 2008). While the cognitive impairment deriving from the acute alcohol intoxication is well-established, the residual effects of alcohol on cognitive performances are less known (Stephens et al., 2008; Stephans and Verster, 2010). Indeed, the effects of alcohol are not limited to the drunkenness experienced after its consumption but include also the next day effects referred to as a hangover. According to van Schrojenstein Lantman et al. (2016, p. 148) “*the alcohol hangover refers to the combination of mental and physical symptoms, experienced the day after a single episode of heavy drinking, starting when blood alcohol concentration approaches zero.*” More in general, hangover is a state of general discomfort that can negatively affect the activities the day after alcohol intoxication. It is a multidimensional phenomenon characterized by a reduction of sleep quantity and quality, alterations in mood, concentration and general well-being, and several symptoms like headache, dizziness, and nausea (Penning et al., 2010).

Previous studies demonstrate that participants in hangover condition have impaired cognitive performance in different tasks (van Schrojenstein Lantman et al., 2017). In particular, next-day impairments were found for short-term/working memory (McKinney and Coyle, 2004, 2007; Howland et al., 2010; Scholey et al., 2019), long-term memory (Verster et al., 2003; McKinney and Coyle, 2004, 2007), sustained attention (Howland et al., 2010; Rohsenow et al., 2010; McKinney et al., 2012), divided attention (Roehrs et al., 1991), selective attention (McKinney et al., 2012; Devenney et al., 2018, 2019), and performance in psychomotor tasks (McKinney and Coyle, 2004; Kruisselbrink et al., 2006; Grange et al., 2016). Moreover, some studies investigated the next-day impairment in real-life situations, showing that participants reported feeling impaired in their working activity (Rohsenow et al., 2006), and that complex daily tasks such as car driving were affected (Laurell and Törnros, 1983; Verster et al., 2014). Although, differences in the methods (e.g., type of tests, amount, and modality of alcohol consumption) gave rise to conflicting evidence in some cases (for a recent review, see, Gunn et al., 2018), it seems quite consolidate that hangover determines a cognitive impairment involving several processes.

Unlike previous studies, which specifically aimed to determine which cognitive functions are impaired by a hangover and to what extent, the present study has the broader aim to investigate whether hangovers can represent a threat to the validity of cognitive research. Indeed, it is well-known that university students are the preferred category of participants to be enrolled in psychological studies, but it is also well-known that students tend to engage in social drinking behaviors (also on weekdays), which cannot be fully controlled by researchers. Given that the next-day effects of alcohol consumption affect the cognitive performances, the hypothesis of the present study is that the recruitment of hungover students might represent a potential bias for cognitive research.

The risk for this bias to occur depends on the actual impairment shown by the hungover students who decide to go to the university to carry out their daily schedule (i.e., thus, excluding those students who refrain from academic activities). Indeed, it is reasonable to assume that a certain percentage of students do not go to the university the day after a party, while the remaining students go to the university, thus being potentially eligible as experimental participants. The present study focuses on the latter category, testing the cognitive performance of students who spontaneously reached the laboratory to be tested the day after a party. The hypothesis of the present study is that the cognitive performance of this sub-sample of hungover students is worse than that of other students who did not consume alcohol, as well as than that of the same students in a non-hangover condition.

## Materials and Methods

### Participants

Thirty-six university students (*M* = 15; *F* = 21) were recruited for this experiment in exchange for academic credits and equally divided into two groups. Their ages ranged from 19 to 36 years (*M* = 22.3; *SD* = 2.6). Participants were contacted *via* social network and email by the researchers. They had normal or corrected to normal vision.

The study was approved by the University of Trieste Ethics Committee (approval n. 80, dd. 12.06.2017), and was conducted in accordance with the recommendations of the Committee. Written informed consent was obtained from each participant before starting the experiment.

### Materials

The experiment was built through the PsychoPy software, version 3.0. The PC was a Dell desk computer with Intel Core i5 (RAM: 4 Gb). The monitor used was a Quato Intelli Proof 242 excellence (24 in), with a 1,024 × 768 resolution. The participants’ responses were collected through a five-button serial response box. For estimating the blood alcohol concentration, a G3 Ferrari Drinkontrol EVO 1YB00200 breathalyzer was used.

Questionnaires were also used. The first part of the questionnaire regarded socio-demographic and personal information (e.g., weight, height, and age), and assessed the participants habits concerning alcohol consumptions. In particular, participants were asked to report how likely they go out and drink more alcohol than usual on nights preceding a working day, for each day, and how likely they would go to the university the day after a weekday party (on a Likert scale from 0 to 7). These scores were then re-coded as follows: 0–1 = very unlikely; 2–3 = unlikely; 4–5 = likely; and 6–7 = very likely. Moreover, information about what they did the night before and, in particular, about the amount (i.e., number of alcohol units) and the kind of alcohol consumed, and the number of hours of sleep were asked. The second part of the questionnaire consisted of the Alcohol Hangover Severity Scale (AHSS; Penning et al., 2013), in which participants were asked to indicate to what extent they were experiencing 12 typical hangover symptoms (e.g., fatigue, concentration problems, and confusion) on a 10-points Likert scale.

### Experimental Design

Participants were divided in two groups. The first group was tested the day after a party (Hangover group), while the second group was tested after a night of alcohol abstinence and normal rest (Non-hangover group). Both groups were tested twice: in the second session – approximately 1 week after the first session – all participants were retested in a non-hangover condition. Thus, a 2 × 2 mixed measures design was employed, with the variable Group (Hangover vs. Non-hangover) between participants and the variable Time (T0 and T1) within participants.

### Procedure

Participants were recruited *via* Facebook through the university groups, and then contacted *via* email by the researchers. They were told they could decide to be tested a first time (T0) either the morning after a party (Hangover condition) or the morning after a night of normal rest without consuming alcohol (Non-hangover condition). All participants were tested a second time (T1) in a non-hangover condition.

Participants were told that they would take part in a research concerning the social habits and lifestyle of University students and their cognitive abilities. They were told that the laboratory was available from 10 AM to 1 PM and were asked to reach the laboratory to be tested on a day of their choice. In this way, they could be tested without an appointment, simply going to the laboratory during their free time (e.g., break between two lectures). Indeed, the laboratory was located in the campus area, where the other academic activities take place.

The experiment took place in a quiet room, without environmental distractions. Participants were asked to complete the informed consent before starting the experiment. They were asked to sit in front of the computer at a distance of approximately 50 cm. They were asked to execute three different cognitive tasks in the following order: parity judgment task, parity judgment task with rule switch, and arithmetic verification task.

#### Task 1: Parity Judgment Task

In this task, participants were exposed to a white fixation point on a gray screen for 500 ms, which then disappeared. After 500 ms, a number (ranging from 1 to 9, excluding 5) was presented. Participants were required to keep their indexes on the keys of the response box and to press the leftmost key if the number was even, or the rightmost key if the number was odd. The stimuli remained on the screen until the participants pressed a key, and after 500 ms a new trial started. Before the experimental session, participants performed a practice session, with two repetitions of the eight stimuli in random order, and after every response a feedback was given about accuracy and response time. The experimental session consisted of five repetitions of the stimuli in random order (40 trials), and no feedback was given. Participants were asked to be as fast and accurate as possible. This task is typically used for research on the Spatial-Numerical Association of Response Codes (SNARC) effect (Dehaene et al., 1993) and implies the retention of the rules (i.e., if odd, press right and if even, press left) in the working memory, the perception of the stimulus and the recognition of its category (odd/even), the selection of the appropriate rule (i.e., if odd, press right vs. if even, press left), and the execution of the motor response.

#### Task 2: Parity Judgment Task With Rule Switch

In this task, the stimuli were exactly the same as the previous task, with the exception of the color of the number, which was either white or black. Like in the previous task, participants were required to press the leftmost key if the number was even or the rightmost key if the number was odd, when the numbers were white. However, when the numbers were black, participants had to reverse the rule; namely, they had to press the leftmost key if the number was odd, or the rightmost key if the number was even. Before the experimental session, participants performed a practice session, with 16 white stimuli (each of the eight digits was presented twice) and eight black stimuli in random order, and after every response, a feedback was given about accuracy and response time. The experimental session consisted of five repetitions of the stimuli used in the practice session (120 trials), and no feedback was given. Participants were asked to be as fast and accurate as possible. It is noteworthy that, in the majority of trials, participants were required to follow the rule which was already consolidated in the previous task, and only in 1/3 of the trials they were required to reverse the keys. Thus, this task implies the retention of the rules (e.g., if odd, press right, if even, press left, and if black reverse the odd-even rule) in the working memory, the perception of the color of the stimulus and the recognition of its category (odd/even), the selection of the appropriate odd-even rule (e.g., if odd, press right vs. if even, press left), the inhibition of the predominant motor response and the reversion of the rule if the stimulus is black, and the execution of the appropriate motor response.

#### Task 3: Arithmetic Verification Task

In this task, participants were exposed to relatively complex mathematical operations and were required to verify whether the result was correct. Participants were required to keep their indexes on the keys of the response box and to press the leftmost key if the displayed result was correct or the rightmost key if the displayed result was incorrect. The stimuli remained on the screen until participants pressed a key, and after 500 ms a new trial started. Before the experimental session, participants performed a practice session, with eight trials (half correct and half incorrect), and after every response feedback was given about accuracy and response time. Then they performed three blocks of 16 trials each, resulting in a total of 48 trials (half correct and half incorrect). The structure of the equations varied depending on the blocks, for instance “(7 × 2)−4 = 9” in the first block, “(31 × 2)−6 = 56” in the second block, and “[(9/3) × 5]−7 = 8” in the third block. Participants were asked to be as fast and accurate as possible. This task implies the retention of the rules (i.e., if correct, press left and if incorrect, press right) in the working memory for the entire duration of the task, the mental calculation of the first operation, the retention of the result in the working memory, and the use of this result to calculate the following operation(s). Finally, participants compared the mentally calculated result with the displayed result, selecting the appropriate rule (if correct, press left vs. if incorrect press right), and executed the motor response.

At the end of the cognitive tasks, all participants were required to complete the questionnaire. Finally, participants were asked to take a breath alcohol test, to verify that they were in a hangover state (blood alcohol concentration <0.02%), and not still under alcohol intoxication.

### Data Analysis

The response times and accuracy for all tasks were analyzed. With regard to response times, before starting the analysis, the response times of incorrect responses were eliminated, and then the median response time for each participant for each condition was calculated. Finally, a set of 2 × 2 mixed ANOVAs (Alcohol × Time) were ran. Contrasts were calculated using *t*-tests. With regard to accuracy, the difference between the number of errors at T0 and that at T1 for each participant was calculated for each task. Then, the Mann-Whitney test was run to compare the effects of the Group variable. As for the questionnaires, the scores obtained for each weekday were compared using the Friedman Test, then the Wilcoxon test was used to calculate the contrasts.

## Results

Participants in the hangover condition reported that the average number of alcohol units they consumed the night before the experiment was 7.42 (SD = 2.83). Assuming that, a standard drink has around 10 g of ethanol and normalizing the consumed alcohol per participants’ weight, the estimated average amount of alcohol per weight consumed the night before the experiment was 1.22 g/kg (*SD* = 0.46). The blood alcohol concentration measured through the breath analysis, the morning of the experiment was zero in all participants. The average score on AHSS reported by participants was 4.11 (*SD* = 1.62). The average number of hours of sleep for the Hangover group was 5.10 (*SD* = 2.57) in T0 and 7.38 (*SD* = 1.37) in T1, while for the Non-hangover group it was 6.97 (*SD* = 1.48) in T0 and 7.08 (*SD* = 1.44) in T1.

### Effects of Hangover on Cognitive Performances

The analyses did not reveal significant differences for the accuracy. At T0, the percentage of errors in the three tasks ranged between 2.64 and 6.78% in the Hangover group, and between 3.34 and 6.71% in the Non-hangover group. At T1, the percentage of errors ranged between 2.78 e and 6.37% in the Hangover group, and between 2.01 and 6.13% in the Non-hangover group.

**Figure 1:**
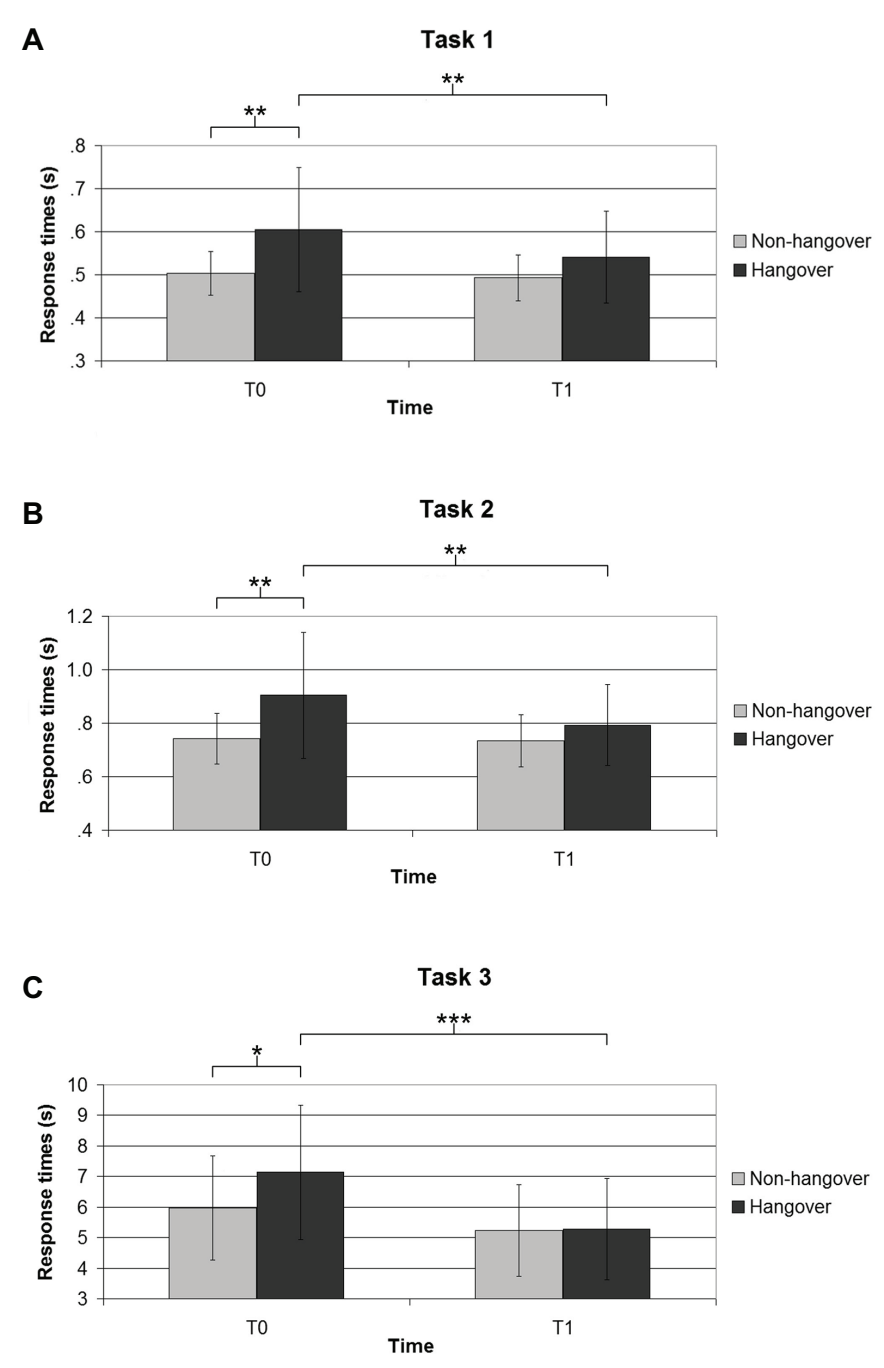
The figure shows the average response times in the three tasks, namely **(A)** parity judgment task; **(B)** parity judgment task with rule switch; and **(C)** arithmetic verification task. For each task, the response times of Hangover and Non-hangover groups are reported, both in T0 and in T1 (when both groups were re-tested in a non-hangover state). Error bars indicate SDs. Significant differences are indicated as follows: ^*^p < 0.05; ^**^p < 0.01; and ^***^p < 0.001.

Instead, significant differences emerged in response times. As for the parity judgment task (task 1 – Figure 1A), the 2 × 2 mixed ANOVA revealed a significant main effect for Group [*F*(1, 34) = 6.33; *p* < 0.005; *η*_p_^2^ = 0.16], for Time [*F*(1, 34) = 8.64; *p* < 0.01; *η*_p_^2^ = 0.20], and a significant interaction [*F*(1, 34) = 4.54; *p* < 0.05; *η*_p_^2^ = 0.12]. In particular, the performances were slower in the Hangover group compared to the Non-hangover group, and in T0 compared to T1. In T0, the Hangover group performed slower than the Non-hangover group [*t*(34) = 2.82; *p* < 0.005; *d* = 0.94], and the Hangover group in T0 performed slower than in T1 [*t*(17) = 2.99; *p* < 0.005; *d* = 0.47], when they were re-tested in a non-hangover state.

Analogously, for the parity judgment with rule switch (task 2 – Figure 1B), a significant main effect for Group [*F*(1, 34) = 5.28; *p* < 0.05; *η*_p_^2^ = 0.13], for Time [*F*(1, 34) = 9.40; *p* < 0.005; *η*_p_^2^ = 0.22], and a significant interaction [*F*(1, 34) = 7.07; *p* < 0.05; *η*_p_^2^ = 0.17] were found. This first analysis was run on all data, independently from the color condition (white or black stimuli, indicating the trials without and with rule switch, respectively). Then the color conditions were separately analyzed, and an analogous pattern of results was found both for white and black trials. Indeed, for white trials (no rule switch), a significant main effect for Group [*F*(1, 34) = 4.88; *p* < 0.05; *η*_p_^2^ = 0.12], for Time [*F*(1, 34) = 10.86; *p* < 0.005; *η*_p_^2^ = 0.24], and a significant interaction [*F*(1, 34) = 4.94; *p* < 0.05; *η*_p_^2^ = 0.13] emerged. For black trials (rule switch), a significant main effect for Group [*F*(1, 34) = 4.81; *p* < 0.05; *η*_p_^2^ = 0.12], for Time [*F*(1, 34) = 4.72; *p* < 0.05; *η*_p_^2^ = 0.12], and a significant interaction [*F*(1, 34) = 6.99; *p* < 0.05; *η*_p_^2^ = 0.17] was found. Similar to the task 1, it was found that the performances were always slower in the Hangover compared to the Non-hangover group, and in T0 compared to T1. The worst performances were obtained by the Hangover group in T0, compared to the Non-hangover group in T0 in all trials [*t*(34) = 2.71; *p* < 0.01; *d* = 0.91], in the white only trials [*t*(34) = 2.58; *p* < 0.01; *d* = 0.87], and in the black only trials [*t*(34) = 2.74; *p* < 0.005; *d* = 0.92], and compared to the same Hangover group when it was re-tested in a non-hangover state (T1), considering all trials [*t*(17) = 3.63; *p* < 0.005; *d* = 0.45], the white only trials [*t*(17) = 3.82; *p* < 0.001; *d* = 0.46], and the black only trials [*t*(17) = 3.34; *p* < 0.005; *d* = 0.45].

Finally, the analysis on response times for the arithmetic verification task (task 3 – Figure 1C) revealed similar results. Apart from the main effect for Group, which was not significant, a significant effect for Time [*F*(1, 34) = 28.94; *p* < 0.001; *η*_p_^2^ = 0.46], and a significant interaction [*F*(1, 34) = 5.42; *p* < 0.05; *η*_p_^2^ = 0.14] emerged. Similar to previous results, it was found that the performances were slower in T0 compared to T1. In T0, the Hangover group performed slower than the Non-hangover group [*t*(34) = 1.77; *p* < 0.05; *d* = 0.59], and the Hangover group in T0 performed slower than in T1 [*t*(17) = 6.20; *p* < 0.001; *d* = 0.88], when they were re-tested in a non-hangover state.

### Alcohol Consumption on Weekdays

**Figure 2:**
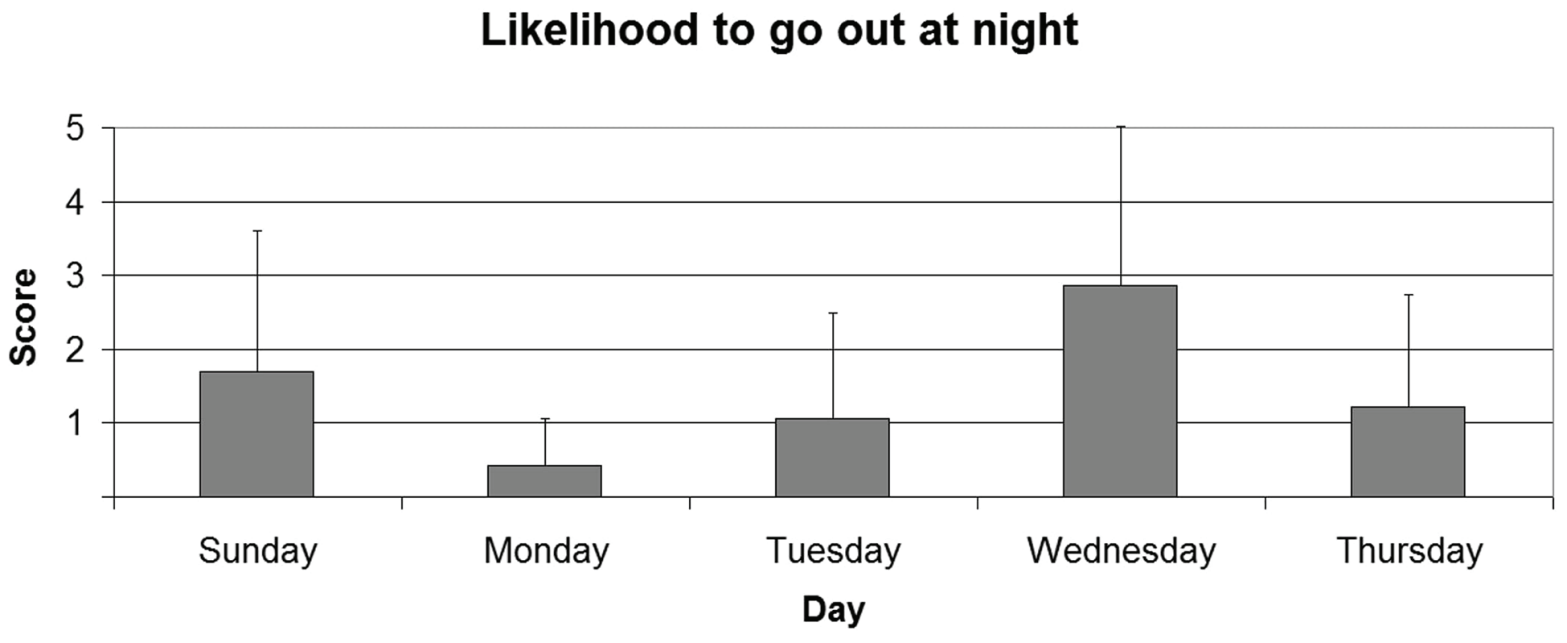
The figure shows the average scores reported by participants to the question “How likely do you go out on the following nights?” on a Likert scale from 0 to 7. Error bars indicate SDs.

The data from the questionnaires revealed that the chance of participants going out and consuming more alcohol than usual on nights preceding a working day (Figure 2) is not equal across all days [*χ*^2^(4) = 47.71; *p* < 0.001]. Indeed, they reported that they are more likely to go out and drink on Wednesday night, compared to Sunday (*Z* = 2.44; *p* < 0.05), Monday (*Z* = 4.40; *p* < 0.001), Tuesday (*Z* = 4.22; *p* < 0.001), and Thursday (*Z* = 3.87; *p* < 0.001) nights. Moreover, the majority of participants (61.1%) reported that it is likely or very likely that they go to the university the day after a party. To further confirm these results, another group of participants were empirically tested to verify the actual presence of hungover students at the university on Thursday (i.e., the day after the highest rated night for parties) and on Tuesday (i.e., the day after a regular weekday) mornings. A random sample of students who were in the university campus from 10 AM to 1 PM on Thursday and Tuesday of the same week was interviewed. Five-hundred students per day were asked the question: “Did you party and drink more alcohol than usual last night?” It was found that the prevalence of hungover students in the campus on Thursday morning was 13.2% (*n* = 66), while the prevalence of hungover students on Tuesday morning was 3.8% (*n* = 19). The data obtained on Thursday and Tuesday were compared by applying a chi-square test, and a statistically significant effect (*χ*^2^ = 28.40; *p* < 0.001) emerged. In order to replicate this finding, the same observations were conducted on the following week, and analogous results were found (*χ*^2^ = 16.95; *p* < 0.001).

## Discussion

There is a vast literature describing the impairments of cognitive functions determined by a hangover. The present study had the broader aim to investigate whether hangover can represent a threat to the validity of cognitive research. Indeed, since a certain percentage of students tend to consume alcohol on weekdays and go to the university the following day, such students are potentially eligible for taking part in psychological experiments, and their participation could affect the results. The hypothesis of the present study was that the cognitive performances of hungover students – who spontaneously reached the laboratory the day after a party – would be somehow impaired. The results confirmed this hypothesis: in all three cognitive tasks, participants in hangover condition performed significantly slower than the non-hangover participants. Moreover, the hungover participants performed significantly faster when re-tested in a non-hangover condition.

Similar to previous studies in the domain of the cognitive effects of a hangover (McKinney and Coyle, 2004; McKinney et al., 2012), the task used revealed a general impairment of the cognitive processes in the hangover condition. In particular, in the parity judgment task, participants in the hangover condition performed slower than the non-hangover participants in T0, and then this difference disappeared in T1, when both groups were re-tested in a non-hangover condition, as suggested by the significant interaction. This means that the hangover affected the cognitive functioning of participants, resulting in slower response times, and that this impairment is reversible when this state ceases. While this task was moderately challenging, the second task was more demanding, since it was the same parity judgment task in which it was required to inhibit the “automatic” response and switch the key in certain trials. The results indicate that, even in this more difficult task, the pattern of responses is analogous. Indeed, similar to the previous task, at T0 the response times of hungover participants were affected compared to the non-hangover participants, while at T1 – when both groups were non-hangover – the cognitive abilities were restored, and the differences disappeared. Finally, in the third task, in which participants were required to mentally execute mathematical calculations, the analyses revealed an analogous pattern of results. Indeed, similar to previous tasks, the interaction suggests that the hangover participants at T0 were slower in executing the mental calculations compared to the non-hangover participants, and this difference disappeared at T1.

It is noteworthy that in all tasks, there was no difference in terms of accuracy, but only in terms of response times. It was somehow expected because in this experiment participants had no temporal constraints, since the stimuli remained on the screen until the participants responded. Consequently, participants favored accuracy over speed so that the impairment given by hangover was revealed only by response times. This pattern can be explained by a well-known phenomenon, the speed-accuracy tradeoff (for a review, see Heitz, 2014). According to this phenomenon, it is possible to predict that by adding a time constraint the effect would be revealed by accuracy instead of by response times.

Notably, three different cognitive tasks characterized by an increasing level of complexity determined the same pattern of results and comparable effect sizes (medium to high). We can speculate that the relatively high level of hangover severity determined a quite high level of impairment, independently of the complexity of the task. Due to the experimental design employed, it was not possible to clearly determine how different levels of hangover severity could affect performances in these tasks. It would be interesting to investigate whether effect size increases/decreases when testing participants with different levels of severity; future studies should address this point.

The hangover effect on response times seems to occur independently of the tasks used, which had a medium to high level of difficulty. Indeed, these tasks were quite complex and involved several cognitive processes, since perceptual, attentional, motor, and memory processes were engaged. In particular, the working memory was probably the most involved process in all three tasks: in task 1, participants had to associate the right rule depending on the parity of the numbers; in task 2, they had to associate the right rule depending on the parity and the color of the numbers; and in task 3, they had to retain the results of mental operations and use them to mentally calculate other operations. Given the involvement of the working memory in all tasks and the consistency of the results across the tasks, the results could be mainly attributed to the temporary impairment of this process. This interpretation is in line with the findings obtained by Howland et al. (2010), who showed the involvement of the working memory in the next-day cognitive effects of alcohol consumption.

The results of the present study suggest that the cognitive effects of alcohol are not limited to the day of alcohol consumption but are still present the next morning. It is noteworthy that the enrolled participants are not representative of all university students who partied the night before, but just of a subsample, namely of those who decided to go to the university the morning after, regardless of their hangover status. Indeed, they were not forced to go to the laboratory to be tested on a specific day, but they could spontaneously reach the laboratory on a day/time of their choice (e.g., on break between two lectures). Surprisingly, the students tested showed a quite high level of hangover severity (AHSS = 4.11), compared to previous studies (Penning et al., 2013; Verster et al., 2014; Howse et al., 2018), suggesting that even a relatively severe hangover did not prevent participants from going to the university and carrying out their scheduled activities. If this generalized to the majority of hungover students, they would be potentially eligible – among all the other students – for psychological experiments and could consequently affect the internal validity of the same experiments. Indeed, if these students were enrolled as participants, the researchers could erroneously attribute the poor cognitive performances of these participants to their experimental manipulation, ignoring a confounding variable such as the hangover.

Another important issue addressed in the present study regards the prevalence of hungover students in the university campus on weekdays. Previous literature anecdotally reported that students typically go out and party also on weekdays, and in particular, on Wednesdays and on Thursdays, depending on the social habits of their city/country (Glindemann et al., 2007; Stephens et al., 2014). The students of the university in which the study took place (i.e., in an Italian context) showed a similar habit, reporting that they use to go out on Wednesday more than any other day (excluding Friday and Saturday). When the prevalence of hungover students in the university campus has been empirically tested, it was found that on Thursday morning (i.e., the day after Wednesday night) the prevalence of hungover students was over three times higher than on Tuesday morning (i.e., the day after Monday night). These results suggest that this phenomenon is not equally distributed across the weekdays, and that on certain days there is a higher chance to recruit hungover students as participants. Thus, the day of testing might enhance the confounding effect of the hangover variable. For instance, a researcher might be interested in investigating the effects of working memory training on a sample of healthy participants, such as university students. It is possible that a group of participants is tested on Tuesday and re-tested the next week on Thursday (or the opposite), after the training: in this case. the results might be negatively (or positively) altered by the hangover variable, leading researchers to wrongly accept (or reject) the null hypothesis. In other words, the observed effects might be due not only to the training, but also to the confounding effects of hangover (which may vary depending on the day of the week, according to the cultural habits of each country).

To prevent future studies in cognitive psychology from being biased by the potential hangover state of participants, researchers should take this aspect into account, measuring it and explicitly reporting how they did so. In this regard, there are two possible options, that is, self-report assessments and blood alcohol concentration monitoring (e.g., breathalyzer and/or wearable technology; Verster et al., 2019). These options are not mutually exclusive, and it would be ideal to always use both; however, in studies whose aim is not to specifically evaluate the effects of alcohol consumption on cognition, including at least a self-report assessment of hangover is recommended. In this regard, the authors’ suggestion is to explicitly ask participants whether they consumed alcohol the night before the experiments, and whether they slept an adequate number of hours. If researchers are informed that one or more participants consumed large amounts of alcohol and slept few hours the night before, they should exclude these participants from the experiments. Moreover, when describing the participants of studies run on university students, the authors’ recommendation is that researchers include this statement: “All participants reported that their psychophysiological state was not affected by alcohol consumption or insufficient sleep in the last 24 h.”

Like every study, the present one has some limitations. Participants were tested in relatively complex tasks, which were mentally demanding, but it is not possible to know whether simpler tasks would be affected as well. Moreover, the prevalence of hungover students in the campus was observed at the end of the semester (just before the exams); consequently these observations might be underestimated. Another limitation is that, although we tried to limit the effects of expectations/motivations of participants in the experiment, we cannot exclude that they could have affected the results.

As for future developments, the present study focused only on cognitive abilities, but it cannot be excluded that an effect of a hangover on other psychological areas might occur. For instance, emotional, and psychosocial factors could also be altered by the state of hangover. Future studies should investigate to what extent hangover could influence similar variables and, consequently, affect the research in these fields. Moreover, other factors that could affect the validity of cognitive experiments could be investigated, such as the reduced sleep typically associated to the compulsive use of internet and social media (Demirci et al., 2015; Cerniglia et al., 2019)

## Conclusion

In conclusion, the results of the present study further confirm that the cognitive performances of healthy participants (i.e., university students) are affected by hangover, and reveal that the prevalence of hungover students in the university campus varies depending on the day of the week. Considering that university students are usually enrolled in experiments investigating the typical functioning of cognitive processes, it is important that researchers ascertain the psychophysiological conditions of the enrolled participants, in order to avoid the results of their experiments from being biased by the effects of potential hangover states. Unless the experiments specifically concern the effects of alcohol consumption on cognitive functions, researchers often do not report whether their participants consumed alcohol the night before the experiments; the lack of this information does not allow to know whether this aspect was neglected, or it was investigated and resulted that no participants had consumed alcohol the night before. Therefore, researchers investigating the typical functioning of cognitive processes are recommended to ask participants about alcohol consumption and/or insufficient sleep the night before the experiment, to exclude those who are in hangover state, and to report that the enrolled participants declared that their psychophysiological state was not affected by hangover.

## Data Availability Statement

All datasets presented in this study are included in the article/Supplementary Material.

## Ethics Statement

The studies involving human participants were reviewed and approved by University of Trieste ethics committee. The patients/participants provided their written informed consent to participate in this study.

## Author Contributions

MM conceived the idea. All authors designed the study. SM and VP programmed the stimuli. SM collected the data. MM and SM prepared the dataset for the analyses. MM, SM, and TA analyzed the data. MM, SM, and FS prepared the first draft of the manuscript. All authors contributed to the article and approved the submitted version.

## Conflict of Interest

The authors declare that the research was conducted in the absence of any commercial or financial relationships that could be construed as a potential conflict of interest.

## References

[ref1] Cerniglia L. Guicciardi M. Sinatra M. Monacis L. Simonelli A. Cimino S. (2019). The use of digital technologies, impulsivity and psychopathological symptoms in adolescence. Behav. Sci. 9:82. 10.3390/bs9080082, PMID: 31344851 PMC6721411

[ref2] Chiandetti C. Pecchia T. Patt F. Vallortigara G. (2014). Visual hierarchical processing and lateralization of cognitive functions through domestic chicks’ eyes. PLoS One 9:e84435. 10.1371/journal.pone.0084435, PMID: 24404163 PMC3880297

[ref3] Corona F. Pau M. Guicciardi M. Murgia M. Pili R. Casula C. (2016). “Quantitative assessment of gait in elderly people affected by Parkinson’s Disease” *in 2016 IEEE International Symposium on Medical Measurements and Applications (MeMeA)*; May 15-18, 2016; 1–6.

[ref4] Dehaene S. Bossini S. Giraux P. (1993). The mental representation of parity and number magnitude. J. Exp. Psychol. 122, 371–396. 10.1037/0096-3445.122.3.371

[ref5] Del Boca F. K. Darkes J. Greenbaum P. E. Goldman M. S. (2004). Up close and personal: temporal variability in the drinking of individual college students during their first year. J. Consult. Clin. Psychol. 72, 155–164. 10.1037/0022-006X.72.2.155, PMID: 15065951

[ref6] Demirci K. Akgönül M. Akpinar A. (2015). Relationship of smartphone use severity with sleep quality, depression, and anxiety in university students. J. Behav. Addict. 4, 85–92. 10.1556/2006.4.2015.010, PMID: 26132913 PMC4500888

[ref7] Devenney L. E. Coyle K. B. Verster J. C. (2018). The impact of expectancy on cognitive performance during alcohol hangover. BMC. Res. Notes 11:730. 10.1186/s13104-018-3827-2, PMID: 30333045 PMC6193303

[ref8] Devenney L. E. Coyle K. B. Verster J. C. (2019). Memory and attention during an alcohol hangover. Hum. Psychopharmacol. Clin. Exp. 34:e2701. 10.1002/hup.2701, PMID: 31297901 PMC6771905

[ref9] Glindemann K. E. Wiegand D. M. Geller E. S. (2007). Celebratory drinking and intoxication: a contextual influence on alcohol consumption. Environ. Behav. 39, 352–366. 10.1177/001391650290949

[ref10] Grange J. A. Stephens R. Jones K. Owen L. (2016). The effect of alcohol hangover on choice response time. J. Psychopharmacol. 30, 654–661. 10.1177/0269881116645299, PMID: 27166364

[ref11] Guicciardi M. Crisafulli A. Doneddu A. Fadda D. Lecis R. (2019). Effects of metabolic syndrome on cognitive performance of adults during exercise. Front. Psychol. 10:1845. 10.3389/fpsyg.2019.01845, PMID: 31440195 PMC6694762

[ref12] Gunn C. Mackus M. Griffin C. Munafò M. R. Adams S. (2018). A systematic review of the next-day effects of heavy alcohol consumption on cognitive performance. Addiction 113, 2182–2193. 10.1111/add.14404, PMID: 30144191 PMC6282576

[ref13] Heitz R. P. (2014). The speed-accuracy tradeoff: history, physiology, methodology, and behavior. Front. Neurosci. 8:150. 10.3389/fnins.2014.00150, PMID: 24966810 PMC4052662

[ref14] Howland J. Rohsenow D. J. Greece J. A. Littlefield C. A. Almeida A. Heeren T.. (2010). The effects of binge drinking on college students’ next-day academic test-taking performance and mood state. Addiction 105, 655–665. 10.1111/j.1360-0443.2009.02880.x, PMID: 20403018 PMC2859622

[ref15] Howse A. D. Hassall C. D. Williams C. C. Hajcak G. Krigolson O. E. (2018). Alcohol hangover impacts learning and reward processing within the medial-frontal cortex. Psychophysiology 55:e13081. 10.1111/psyp.13081, PMID: 29600513

[ref16] Koelega H. S. (1995). Alcohol and vigilance performance: a review. Psychopharmacology 118, 233–249. 10.1007/BF02245951, PMID: 7617815

[ref17] Kruisselbrink L. D. Martin K. L. Megeney M. Fowles J. R. Murphy R. J. L. (2006). Physical and psychomotor functioning of females the morning after consuming low to moderate quantities of beer. J. Stud. Alcohol 67, 416–420. 10.15288/jsa.2006.67.416, PMID: 16608151

[ref18] Laurell H. Törnros J. (1983). Investigation of alcoholic hangover effects on driving performance. Blutalkohol 20, 489–499.

[ref19] McKinney A. Coyle K. (2004). Next day effects of a normal night’s drinking on memory and psychomotor performance. Alcohol Alcohol. 39, 509–513. 10.1093/alcalc/agh099, PMID: 15477234

[ref20] McKinney A. Coyle K. (2007). Next-day effects of alcohol and an additional stressor on memory and psychomotor performance. J. Stud. Alcohol Drugs 68, 446–454. 10.15288/jsad.2007.68.446, PMID: 17446985

[ref21] McKinney A. Coyle K. Penning R. Verster J. C. (2012). Next day effects of naturalistic alcohol consumption on tasks of attention. Hum. Psychopharmacol. 27, 587–594. 10.1002/hup.2268, PMID: 24446537

[ref22] O’Malley P. M. Johnston L. D. (2002). Epidemiology of alcohol and other drug use among American college students. J. Stud. Alcohol S14, 23–39. 10.15288/jsas.2002.s14.23, PMID: 12022728

[ref23] Penning R. McKinney A. Bus L. D. Olivier B. Slot K. Verster J. C. (2013). Measurement of alcohol hangover severity: development of the alcohol hangover severity scale (AHSS). Psychopharmacology 225, 803–810. 10.1007/s00213-012-2866-y, PMID: 23007602

[ref24] Penning R. van Nuland M. Fliervoet L. A. L. Olivier B. Verster J. C. (2010). The pathology of alcohol hangover. Curr. Drug Abuse Rev. 3, 68–75. 10.2174/1874473711003020068, PMID: 20712596

[ref25] Roehrs T. Yoon J. Roth T. (1991). Nocturnal and next-day effects of ethanol and basal level of sleepiness. Hum. Psychopharmacol. Clin. Exp. 6, 307–311. 10.1002/hup.470060407

[ref26] Rohsenow D. J. Howland J. Arnedt J. T. Almeida A. B. Greece J. Minsky S.. (2010). Intoxication with bourbon versus vodka: effects on hangover, sleep, and next-day neurocognitive performance in young adults. Alcohol. Clin. Exp. Res. 34, 509–518. 10.1111/j.1530-0277.2009.01116.x, PMID: 20028364 PMC3674844

[ref27] Rohsenow D. J. Howland J. Minsky S. J. Arnedt J. T. (2006). Effects of heavy drinking by maritime academy cadets on hangover, perceived sleep, and next-day ship power plant operation. J. Stud. Alcohol 67, 406–415. 10.15288/jsa.2006.67.406, PMID: 16608150

[ref28] Ryback R. S. (1971). The continuum and specificity of the effects of alcohol on memory: a review. Q. J. Stud. Alcohol 32, 995–1016. 10.15288/qjsa.1971.32.995, PMID: 4944697

[ref29] Scholey A. Benson S. Kaufman J. Terpstra C. Ayre E. Verster J. C.. (2019). Effects of alcohol hangover on cognitive performance: findings from a field/internet mixed methodology study. J. Clin. Med. 8:440. 10.3390/jcm8040440, PMID: 30935081 PMC6518120

[ref30] Schweizer T. A. Vogel-Sprott M. (2008). Alcohol-impaired speed and accuracy of cognitive functions: a review of acute tolerance and recovery of cognitive performance. Exp. Clin. Psychopharmacol. 16, 240–250. 10.1037/1064-1297.16.3.240, PMID: 18540784

[ref31] Stephans R. Verster J. C. (2010). The importance of raising the profile of alcohol hangover research. Curr. Drug Abuse Rev. 3, 64–67. 10.2174/1874473711003020064, PMID: 20822482

[ref32] Stephens R. Grange J. A. Jones K. Owen L. (2014). A critical analysis of alcohol hangover research methodology for surveys or studies of effects on cognition. Psychopharmacology 231, 2223–2236. 10.1007/s00213-014-3531-4, PMID: 24633471

[ref33] Stephens R. Ling J. Heffernan T. M. Heather N. Jones K. (2008). A review of the literature on the cognitive effects of alcohol hangover. Alcohol Alcohol. 43, 163–170. 10.1093/alcalc/agm160, PMID: 18238851

[ref34] van Schrojenstein Lantman M. Mackus M. van de Loo A. J. A. E. Verster J. C. (2017). The impact of alcohol hangover symptoms on cognitive and physical functioning, and mood. Hum. Psychopharmacol. Clin. Exp. 32:e2623. 10.1002/hup.2623, PMID: 28750479 PMC5638093

[ref35] van Schrojenstein Lantman M. van de Loo A. J. A. E. Mackus M. Verster J. C. (2016). Development of a definition for the alcohol hangover: consumer descriptions and expert consensus. Curr. Drug Abuse Rev. 9, 148–154. 10.2174/1874473710666170216125822, PMID: 28215179

[ref36] Verster J. C. Bervoets A. C. de Klerk S. Vreman R. A. Olivier B. Roth T.. (2014). Effects of alcohol hangover on simulated highway driving performance. Psychopharmacology 231, 2999–3008. 10.1007/s00213-014-3474-9, PMID: 24563184

[ref37] Verster J. C. van de Loo A. J. Adams S. Stock A. -K. Benson S. Scholey A.. (2019). Advantages and limitations of naturalistic study designs and their implementation in alcohol hangover research. J. Clin. Med. 8:2160. 10.3390/jcm8122160, PMID: 31817752 PMC6947227

[ref38] Verster J. C. van Duin D. Volkerts E. R. Schreuder A. H. C. M. L. Verbaten M. N. (2003). Alcohol hangover effects on memory functioning and vigilance performance after an evening of binge drinking. Neuropsychopharmacology 28, 740–746. 10.1038/sj.npp.1300090, PMID: 12655320

